# Agreement of High-Definition Oscillometry (HDO) and Invasive Blood Pressure Measurements at a Metatarsal Artery in Isoflurane-Anaesthetised Horses

**DOI:** 10.3390/ani12030363

**Published:** 2022-02-02

**Authors:** Lara Twele, Stephan Neudeck, Julien Delarocque, Nicole Verhaar, Julia Reiners, Mike Noll, Julia Tünsmeyer, Sabine B. R. Kästner

**Affiliations:** 1 Clinic for Horses, University of Veterinary Medicine Hannover, Foundation, Bünteweg 9, 30559 Hannover, Germany; julien.delarocque@tiho-hannover.de (J.D.); nicole.verhaar@tiho-hannover.de (N.V.); 2 Small Animal Clinic, University of Veterinary Medicine Hannover, Foundation, Bünteweg 9, 30559 Hannover, Germany; stephan.neudeck@tiho-hannover.de (S.N.); julia.tuensmeier@tiho-hannover.de (J.T.); 3 AniCura Duisburg-Asterlagen Gmbh, Dr.-Detlev-Karsten-Rohwedder-Str. 11, 47228 Duisburg, Germany; julia.reiners@anicura.de; 4 Evidensia Tierärztliche Klinik für Kleintiere Norderstedt GmbH, Kabels Stieg 41, 22850 Norderstedt, Germany; M.Noll@tierklinik-norderstedt.de

**Keywords:** equine, anaesthesia, monitoring, blood pressure, high-definition oscillometry, HDO, metatarsal artery

## Abstract

**Simple Summary:**

In equine anaesthesia, valid and reliable blood pressure monitoring is crucial for adequate blood pressure management. Various non-invasive blood pressure monitors have been studied with varying results. High-definition oscillometry (HDO) promises greater accuracy than conventional oscillometric devices. With the cuff placed at the tail, the monitor gives satisfactory readings in normotensive anaesthetised horses, while for measuring low and high blood pressure, reliability was inadequate. To date, high-definition oscillometry has not been evaluated at the easily accessible equine metatarsal area. Therefore, the objective of this study was to assess agreement between HDO and invasive blood pressure, both obtained at a metatarsal artery at different blood pressure ranges in anaesthetised horses. Additionally, compliance with the American College of Veterinary Internal Medicine consensus guidelines was assessed. Agreement of HDO and invasive blood pressure was acceptable for mean arterial blood pressure during normotension only. During hypotension and hypertension, measurements were not valid. The monitor failed to meet most of the consensus validation criteria. Consequently, invasive blood pressure measurement remains preferable in haemodynamically unstable patients.

**Abstract:**

High-definition oscillometry (HDO) over the metatarsal artery (MA) in anaesthetised horses has not yet been evaluated. This study aimed to assess agreement between HDO and invasive blood pressure (IBP) at the metatarsal artery, and to evaluate compliance with the American College of Veterinary Internal Medicine (ACVIM) consensus guidelines. In this experimental study, 11 horses underwent general anaesthesia for an unrelated, terminal surgical trial. Instrumentation included an IBP catheter in one and an HDO cuff placed over the contralateral MA, as well as thermodilution catheters. Systolic arterial pressure (SAP), mean arterial pressure (MAP), diastolic arterial pressure (DAP), and cardiac output were measured simultaneously. Normotension (MAP 61–119 mmHg) was maintained during the surgical study. Subsequently, hypotension (MAP ≤ 60 mmHg) and hypertension (MAP ≥ 120 mmHg) were induced pharmacologically. For MAP, the agreement between HDO and IBP was acceptable during normotension, while during hypotension and hypertension, IBP was overestimated and underestimated by HDO, respectively. The monitor failed to meet most ACVIM validation criteria. Consequently, if haemodynamic compromise or rapid blood pressure changes are anticipated, IBP remains preferable.

## 1. Introduction

Determination of cardiac output (CO) as a good indicator for blood flow and perfusion is still laborious. Thus, in clinical settings, invasive blood pressure (IBP) measurements in peripheral arteries are well-accepted surrogates for CO measurements. The mean arterial blood pressure (MAP) is dependent on CO and systemic vascular resistance (SVR) as a measure of vessel tone. Valid IBP measurement requires technical equipment and expertise, and the establishment of arterial access can be time consuming.

Various non-invasive blood pressure (NIBP) monitors have been studied in horses at different anatomical locations and with varying results regarding accuracy and precision [1,2,3]. High-definition oscillometry (HDO) is claimed to be more accurate than standard oscillometric devices because of the real-time analysis of pulse amplitudes and precision deflation valves [4].

The performance of the HDO monitor with the cuff placed over the coccygeal artery was assessed in anaesthetised horses [5,6]. In dorsally recumbent horses, the monitor demonstrated the best agreement with IBP in the facial artery (FA) for systolic arterial pressure (SAP) and MAP during normotension but underestimated and overestimated pharmacologically induced hypertension and hypotension, respectively [5]. In laterally recumbent, normotensive horses, MAP, SAP, and diastolic arterial pressure (DAP) were slightly overestimated by HDO [6]. Overall, variability of HDO measurements was greater when total intravenous anaesthesia (TIVA) was used, compared with inhalation anaesthesia [6]. In anaesthetised dogs [7,8] and cats [9], HDO demonstrated poor precision and overestimation of IBP during periods of hypotension.

In adult anaesthetised horses, NIBP measurement in the metatarsal region did not seem to be an appropriate alternative to IBP measurement [1,2], but a better agreement of IBP and NIBP with the cuff placed at the limbs, compared with the tail, was reported in dorsally recumbent horses [1].

To the authors’ knowledge, studies evaluating the HDO monitor over the metatarsal artery (MA) in anaesthetised horses are lacking. Changes in wave reflections from more centrally to peripherally located arteries lead to amplification of pulse waves [10]. Therefore, the aim of this study was to investigate the agreement of IBP and HDO, both obtained at a MA during normotension and pharmacologically induced hypotension and hypertension. Moreover, to assess the possible influence of changes in SVR on arterial oscillations and, thus, HDO performance [1], CO and SVR were determined. Additionally, we aimed to assess compliance with the American College of Veterinary Internal Medicine (ACVIM) consensus guidelines [11]. It was hypothesised that HDO would provide acceptable agreement with IBP and meet ACVIM criteria.

## 2. Materials and Methods

### 2.1. Animals

This study was part of an unrelated, terminal surgical trial investigating ischaemic postconditioning at the equine jejunum. All procedures were reviewed and approved by the Ethics Committee for Animal Experiments of Lower Saxony (Lower Saxony State Office for Consumer Protection and Food Safety (approval number 33.19-42502-04-18/2856).

An a priori power analysis revealed that 20 horses would be necessary to detect a mean difference of 10 mmHg (standard deviation 15 mmHg) between two blood pressure measurement methods [11], with a power of 0.8 (1–β) and α of 5%.

In total, 16 horses were designated for the surgical trial. Measurements of 5 horses were excluded due to instrumentational or technical problems. Data of the remaining 11 horses (7 Warmbloods, 1 Standardbred, and 3 Thoroughbreds; 6 females and 5 males, with a median (range) age of 6 years (4–24 years) and a median (range) body weight of 542 kg (464–596 kg)) were obtained and analysed. The horses had no recent history of cardiovascular disease. Physical examination and routine haematological and biochemical blood examination were without abnormalities. All horses suffered from a chronic, untreatable orthopaedic disease. Therefore, they were euthanised by intravenous (IV) injection of pentobarbitone 80 mg/kg IV (Release, WDT, Garbsen, Germany) at the end of the study, without regaining consciousness, and subsequently used for educational purposes.

### 2.2. Anaesthesia Protocol and Monitoring

Food but not water was withheld for six hours prior to anaesthesia. All horses were premedicated with xylazine 0.7 mg/kg IV (Xylavet, CP-Pharma, Burgdorf, Germany). Induction of anaesthesia was performed by ketamine 2.2 mg/kg IV (Narketan, Vetoquinol, Ismaning, Germany) and diazepam 0.1 mg/kg IV (Ziapam, Ecuphar, Greifswald, Germany). Following orotracheal intubation, the animals were hoisted to a padded air mattress, positioned in dorsal recumbency, and connected to a large animal anaesthesia machine (Vet.-Tec. Model LAVC 2000, J.D. Medical Distributing Company, Phoenix, AZ, USA) via a circle breathing system. Anaesthesia was maintained with isoflurane in 100% oxygen (Isofluran CP, CP-Pharma, Burgdorf, Germany). Pressure cycled mechanical ventilation with positive inspiratory pressure (PIP) of 20–25 cmH_2_O was initiated. Respiratory rate and PIP were adjusted to maintain an end-tidal carbon dioxide (PE’CO_2_) between 4.7 and 6 kPa (35–45 mmHg). Standard anaesthetic monitoring included sidestream capnography, a lead II electrocardiogram, and inspiratory and end-expiratory gas concentrations displayed on a multiparameter monitor (Cardiocap 5 monitor, Datex-Ohmeda, Freiburg, Germany). The gas analyser was calibrated before each experiment by a 2-point calibration at expected concentrations (Quick Cal calibration gas, Datex, Helsinki, Finland).

### 2.3. Instrumentation

For IBP measurement, horses were equipped with a 20 gauge over-the-needle-catheter (Venocan PLUS, Kruuse; Langeskov; Denmark) that was inserted into one MA and connected to a pre-calibrated disposable electronic pressure transducer (DTXPlus, Becton Dickinson, Heidelberg, Germany) via a non-compliant fluid-filled (10 IU/mL heparinised saline; Heparin-Natrium-5000-ratiopharm, Ratiopharm, Ulm, Germany) extension line. The pressure transducer was positioned at the level of the shoulder point and zeroed to atmospheric pressure. A fast-flush test with visual evaluation of the arterial pressure waveform was performed to confirm the presence of a square wave and two oscillations at the beginning of the experiment and at a regular interval to avoid excessive overdamping or underdamping and clot formation.

For the NIBP measurement, a commercially available HDO equine cuff (width 8 cm) (S+B medVet, Babenhausen, Germany) was placed over the palpable MA around the contralateral cannon bone at the same height as the IBP catheter. The cuff was connected to the HDO monitor Memo Diagnostic Equine (S+B medVet, Babenhausen, Germany). Readings and oscillometric curves were visible in real time on a tablet device using the manufacturer’s software (VetHDO 2.7.1.2, S+B medVet, Babenhausen, Germany). To account for the effects of hydrostatic pressure, the vertical distance between the middle of the HDO cuff and the level of the xiphoid process was measured by a tape measure, and a correction factor (1 cm~0.74 mmHg) was applied [5]. This value was then added to the pressures (MAP, DAP, SAP) measured by the HDO device.

For CO measurements, two 8 Fr catheter introducers (Argon Exacta, Argon Medical Devices, The Hague, The Netherlands) were aseptically placed in the right jugular vein. A thermistor-tipped thermodilution catheter (Argon CritiCath, Argon Medical Devices, The Hague, The Netherlands) with a length of 110 cm was placed into the pulmonary artery (PA), and a second balloon-tipped catheter was placed into the right atrium (RA). Correct placement was confirmed by visual inspection of the pressure waveforms.

### 2.4. Blood Pressure and Cardiac Output Measurements

After successful instrumentation, blood pressure (IBP and HDO obtained at the MA) and CO measurements were performed simultaneously, representing a measurement time point.

The HDO measurement was started manually, and at the end of each HDO cycle, all readings (MAP, DAP, SAP, and heart rate) obtained simultaneously by HDO and IBP were recorded.

Cardiac output measurements were performed by the bolus thermodilution method. A bolus of iced 0.9% sodium chloride solution (50 mL) was injected manually into the RA, while the three closest CO values of a maximum of five injections were used for further analysis. For calculation of cardiac index (CI) and systemic vascular resistance index (SVRI) standard equations were used (Table S1). Calculated CI and SVRI were related to IBP obtained at the MA.

All data were manually recorded in an electronic spreadsheet and used for data analysis. Erroneous NIBP measurements (displayed by the HDO software as ‘error code’) were not considered for statistical analysis. Additionally, retrospective visual evaluation and graduation into ‘good’ and ‘poor’ quality oscillometric curves recorded by HDO software were performed by one author, without excluding any of these measurements from data analysis. Good quality oscillometric curves did not contain any artifacts and were characterised by a linear deflation curve and a bell-shaped oscillation curve.

### 2.5. Variation of Blood Pressure

Throughout the surgical study, normotension (MAP 61–119 mmHg) was achieved by infusion of Lactated Ringer’s solution (5–10 mL/kg/h; Ringer-Laktat-Lösung, B. Braun Melsungen, Germany) and dobutamine (0.3–1.2 µg/kg/min; Dobutamine-ratiopharm, Ratiopharm, Ulm, Germany). The duration of the surgical study was approximately 210 min. After the termination of the surgical trial, the manipulation of blood pressure was initiated, allowing a short stabilisation period of 5 min at each range. To achieve hypotension (MAP ≤ 60 mmHg), inspiratory isoflurane concentration was increased, while infusions (crystalloids and dobutamine) were discontinued. Hypertension (MAP ≥ 120 mmHg) was induced by decreasing inspiratory isoflurane concentration and resuming blood pressure support by crystalloid infusions and dobutamine (2.7–3.0 µg/kg/min). To further increase blood pressure, additional norepinephrine (0.15–0.9 µg/kg/min; Arterenol, Sanofi Aventis, Frankfurt, Germany) was administered.

### 2.6. Statistical Analysis

A random selection by R statistical software [12] of three measurements was made to avoid sampling bias in cases in which more than three consecutive blood pressure measurements were recorded per measurement time point. For further analysis, those three measurements were averaged.

Repeated-measures correlation or within-participants correlation [13] was estimated for each comparison of the two measurement methods using the ‘rmcorr’ R package [14]. A Bland–Altman analysis was conducted using a linear mixed model to calculate the limits of agreement (LOA), as described in [15]. Briefly, the ‘nlme’ R package [16] was used to fit a random slope model by method nested within the horse. Standard deviations were estimated per stratum of the method. Model assumptions were checked by visual inspection of a scatterplot of fitted values against standardised residuals. The resulting LOA values were based on the standard deviation of the difference between a pair of measurements by the two methods. The bias was reported as the mean difference between NIBP and IBP. A positive bias reflected underestimation and a negative bias reflected overestimation of IBP by NIBP. The level of significance was *p* ≤ 0.05.

Calculations were performed using R statistical software [12]. Bland–Altman graphs, the XY plot, and plots for CI and SVRI in relation to blood pressure were drawn by GraphPad Prism (GraphPad Software, version 8.4.1, San Diego, CA, USA).

For comparison of our data with the ACVIM guidelines, the mean difference and standard deviation between paired measurements were calculated, using a standard spreadsheet program (Microsoft Excel, Redmond, WA, USA).

## 3. Results

**Figure 1:**
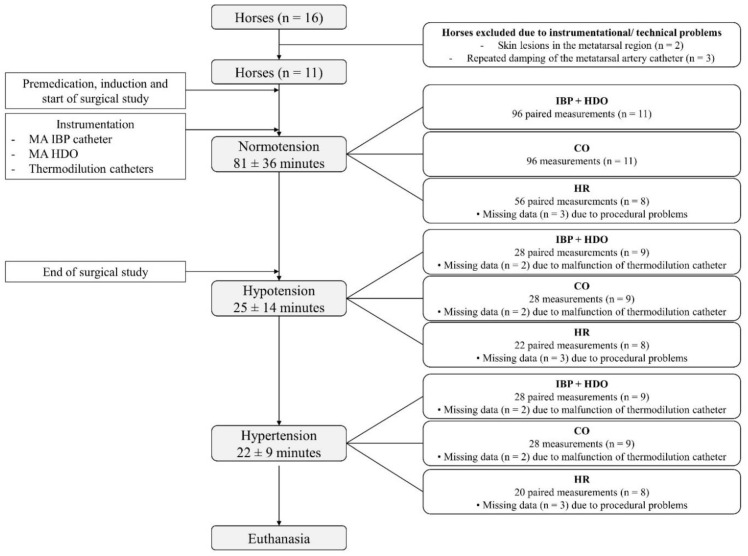
Consolidated Standards for the Reporting of Trials (CONSORT) flow diagram. Of 16 horses included in the terminal surgical trial, 5 horses did not meet inclusion criteria due to instrumentational or technical problems. Data of the remaining 11 horses were obtained and analysed. Invasive blood pressure (IBP), blood pressure obtained by high-definition oscillometry (HDO), cardiac output (CO), and heart rate (HR) were recorded simultaneously throughout the study. For normotension (mean arterial blood pressure (MAP) 61–119 mmHg), hypotension (MAP ≤ 60 mmHg), and hypertension (MAP ≥ 120 mmHg), the mean (± standard deviation) duration of measurements is given. The number of data recorded and included in the statistical analysis, as well as the number of missing data, is shown. Reasons for missing data were instrumentational, procedural, or technical issues. *n* = number of horses, IBP = invasive blood pressure, HDO = high-definition oscillometry, MA = metatarsal artery, MAP = mean arterial pressure.

In total, 152 paired measurements (*n* = 11 horses) for cardiac output and blood pressure obtained simultaneously by IBP and HDO at the MA were analysed. More data were recorded during normotension (96 paired measurements), compared with hypotension (28 paired measurements) and hypertension (28 paired measurements). An overview of data selection for statistical analysis, duration of different measurement periods, and exclusion criteria of data is depicted in Figure 1.

### 3.1. Blood Pressure Measurements and Heart Rate

**Figure 2:**
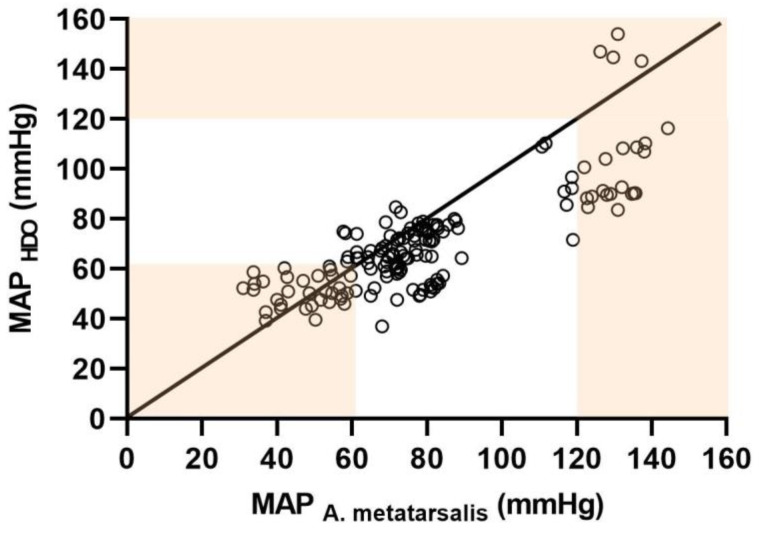
XY plot for mean arterial pressure (MAP) obtained by invasive (IBP) technique at the metatarsal artery (MA) (x-axis) and non-invasive (NIBP) technique by high-definition oscillometry (HDO) (y-axis) at the contralateral MA in 11 isoflurane anaesthetised and dorsally recumbent horses at different blood pressure ranges (normotension: MAP 61–119 mmHg; hypotension: MAP ≤ 60 mmHg; hypertension: MAP ≥ 120 mmHg). A total of 152 paired measurements are depicted. The black straight line represents the line of identity of both measurement techniques. The red-coloured zones represent hypotension and hypertension, respectively.

**Figure 3:**
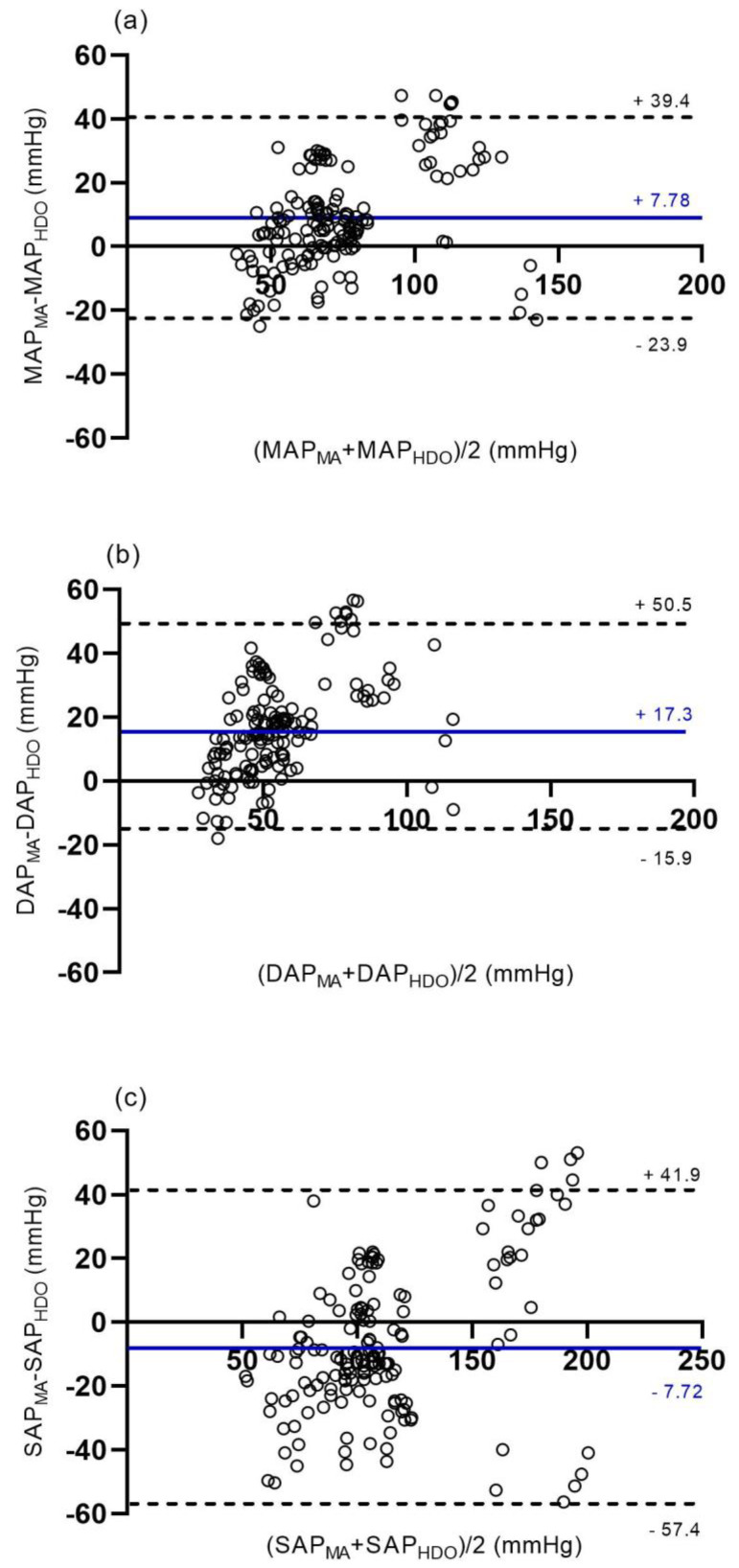
Bland–Altman plots for (**a**) mean arterial pressure (MAP), (**b**) diastolic arterial pressure (DAP), and (**c**) systolic arterial pressure (SAP) measured by invasive (IBP) technique at the metatarsal artery (MA) and non-invasive (NIBP) technique by high-definition oscillometry (HDO) at the contralateral MA in 11 isoflurane anaesthetised and dorsally recumbent horses at different blood pressure ranges (normotension: MAP 61–119 mmHg; hypotension: MAP ≤ 60 mmHg; hypertension: MAP ≥ 120 mmHg). A total of 152 paired measurements are depicted. The blue horizontal line represents mean bias, and the dashed black lines the upper and lower limits of agreement (LOA), respectively. On the x-axis, the average, and on the y-axis, the difference between the two measurements is shown. A positive bias reflects underestimation, and a negative bias reflects overestimation of IBP by HDO.

For MAP the XY plot in Figure 2 illustrates blood pressure measurements obtained at the MA by IBP and HDO (152 paired measurements). The overall agreement of MAP, DAP, and SAP obtained at the MA by IBP and HDO (152 paired measurements) is depicted by Bland–Altman plots (Figure 3).

MAP was overestimated by HDO during hypotension. Under normotensive conditions, HDO slightly underestimated IBP, while during hypertension, IBP was markedly underestimated by HDO (Figure 2 and Figure 3a)

Particularly during normotension and hypertension, DAP was underestimated by HDO, while SAP was overestimated in hypotension and underestimated in hypertension (Figure 3b,c).

During normotension (96 paired measurements), bias and upper and lower LOA between IBP and HDO for MAP, DAP, and SAP were 8.1 mmHg (−14.1–30.2), 26.8 mmHg (−4.2–57.8), and −9.5 mmHg (−41.1–22.2), respectively.

For the hypotensive blood pressure state (28 paired measurements), bias and upper and lower LOA between IBP and HDO for MAP, DAP, and SAP were −3.5 mmHg (−25.3–18.2), 2.6 mmHg (−15.8–21.0), and −20.1 mmHg (−50.7–10.5), respectively.

During hypertension (28 paired measurements), bias and upper and lower LOA between IBP and HDO for MAP, DAP, and SAP were 25.0 mmHg (−14.5–64.5), 39.1 mmHg (−1.9–76.4), and 12.8 mmHg (−60.5–86.2), respectively.

The median heart rate (range) of all horses was 35 (29–81) beats/minute. Arrhythmias were not observed in any horse. Agreement between invasively and non-invasively measured heart rate was good (bias −0.29 beats/minute, upper-lower LOA −9.6–8.9 beats/minute).

### 3.2. Relationship of CI and SVRI and Blood Pressure

**Figure 4:**
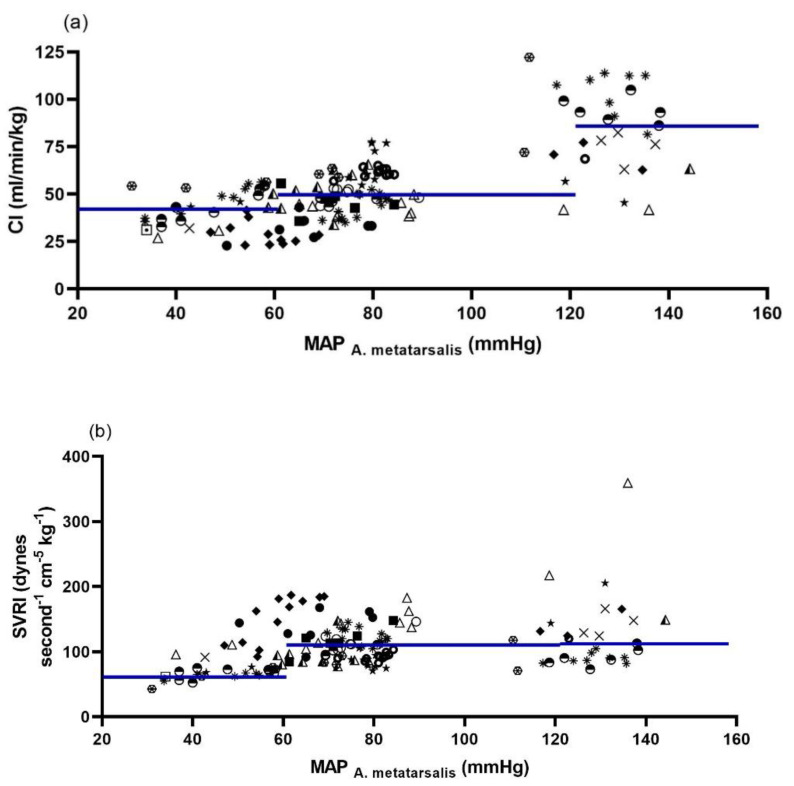
Plots depicting calculated cardiac index (CI) (**a**) and systemic vascular resistance index (SVRI) (**b**) in relation to invasively measured blood pressure (IBP) obtained at the metatarsal artery (MA) in 11 isoflurane anaesthetised and dorsally recumbent horses at different blood pressure ranges (normotension: MAP 61–119 mmHg; hypotension: MAP ≤ 60 mmHg; hypertension: MAP ≥ 120 mmHg). Each symbol represents measurements obtained from one horse. The solid blue horizontal lines represent mean CI and SVRI (*n* = 11 horses) for each blood pressure range, respectively. MAP = mean arterial pressure.

Calculated CI and SVRI in relation to IBP obtained at the MA are shown in Figure 4. Compared with normotension, during hypotension, a decrease in CI and SVRI was present. In the hypertensive blood pressure state, only CI increased, while SVRI was comparable to normotension.

### 3.3. Compliance with ACVIM Guidelines

When applying the 2007 ACVIM standards to the comparison of IBP and HDO measured MAP, DAP, and SAP obtained at the MA, the criteria were fulfilled neither for SAP nor for DAP in this study. For MAP, only a few criteria met the ACVIM standards (Table 1).

### 3.4. Retrospective Assessment of Oscillation Curve Quality

Due to technical problems concerning the HDO software, data of four horses were not available for retrospective evaluation. A total of 25 single (*n* = 7 horses) erroneous (‘error code’) measurements were observed evenly across all blood pressure states. A retrospective visual assessment revealed a total of 84 ‘good’ quality (*n* = 7 horses) and 24 ‘poor’ quality (*n* = 5 horses) oscillometric curves. Poor quality measurements were observed in hypotensive and low normotensive blood pressure ranges.

## 4. Discussion

In this study, HDO placed over a metatarsal artery was able to provide acceptable agreement with IBP for MAP in the normotensive range only and partly met the 2007 ACVIM standards also for MAP only.

Our findings are in accordance with a previous study in which agreement of HDO with IBP for MAP was good during normotension (192 paired measurements) [5]. However, comparable to our study, relatively wide limits of agreement (bias 0.5, LOA −27 to 28 mmHg) were observed, and at low (23 pairs) and high (30 pairs) blood pressures, HDO also overestimated and underestimated IBP, respectively. As suggested by the current and two previous studies in horses [5,6], HDO seems to provide reliable measurements of MAP in normotension only. Nevertheless, early detection of critical blood pressure changes is crucial for clinical decision making in anaesthesia. At low blood pressures, HDO overestimated IBP, meaning that hypotension may go unnoticed. Delayed recognition and treatment of hypotension with possible subsequent hypoperfusion and decreased tissue oxygen delivery may have major consequences such as myopathy [17] or reduced gastrointestinal perfusion [18]. At hypertensive blood pressures, IBP was underestimated by HDO meaning that severe hypertension may also not be detected.

In this study, overall, DAP was underestimated by HDO, while SAP was underestimated at hypertension and overestimated at hypotension. For SAP, these findings are in accordance with the study by [5]. For oscillometric devices, it was shown that the accuracy of DAP and SAP estimates is affected by alterations in arterial wall viscoelastic properties and pressure pulse amplitudes [19]. Both factors may have prevented HDO from adequate estimations of SAP and DAP despite direct pressure waveform analysis. Consequently, using HDO monitors, MAP seems to be the most reliable parameter [20].

Another reason for the inaccuracy of HDO measurements might be the characteristics of the anatomic location itself. As proposed by [2], the geometry of the cannon bone, the depth of the artery, and the compressibility of the surrounding tissues in adult horses may have hampered signal quality.

Appropriate cuff width relative to the circumference of the leg or tail is crucial for the accuracy of NIBP measurement [2,6,21]. Undersized cuffs rather overestimate blood pressure, whereas wide cuffs lead to underestimation of NIBP readings [22]. A cuff width-to-tail and limb-girth ratio of approximately 40% provided the most accurate results in conventional oscillometric devices [21] as well as HDO [6]. The same cuff width and position (8 cm cuff placed over the MA) in another study led to the underestimation of MAP in normotensive anaesthetised horses [2]. In our study, only one cuff size was available which may be regarded as a limitation. However, although we did not numerically determine cuff width to metatarsal circumference ratio in each individual horse, the horses were homogenous in body conformation, and visual evaluation estimated a ratio of approximately 40%.

Hypotension resulted from a reduction in CI and SVRI in most horses. The most probable reason for the reduction in CO is the cessation of inotropic support by dobutamine and fluid support. Additionally, isoflurane enhances myocardial depression and peripheral vasodilation in a dose-dependent fashion, while endotoxaemia caused by surgical intervention may also have played a role. Changes in vasomotor tone may alter the performance of NIBP monitors [1], which was also shown for HDO in anaesthetised dogs [23]. The low arterial tone in combination with release in occlusion pressure might have resulted in an increase in arterial oscillations and, consequently, falsely high amplitudes, resulting in an overestimation of blood pressure by HDO. Therefore, ‘poor’ quality oscillation curves might be an additional indicator for hypotension.

Hypertension mainly resulted from increased CI caused by positive inotropic effects of dobutamine and concomitant reduction in afterload. Due to insufficient raise in MAP by dobutamine alone, additional norepinephrine was administered in all horses. Norepinephrine is considered a potent vasopressor, but it also increases myocardial contractility due to beta-adrenergic stimulation [24]. Since SVRI nearly remained unchanged despite increased MAP, it may be assumed that endotoxaemia caused a decreased response to norepinephrine-induced vasoconstriction, or that the dose of norepinephrine was too low to exert sustainable vasopressor effects. In dogs, it was shown that hypertension and high SVR markedly altered HDO performance [23]. However, our findings suggest that poor HDO performance in hypertension is not depending solely on SVR.

When applying the 2007 ACVIM standards, in this study, the required criteria were not met for SAP and DAP and only partly for MAP. These findings might result from a large variability of the measurements, affecting accuracy and reliability. Similar observations were made in previous studies in anaesthetised horses [21,25]. Only one study succeeded in meeting the ACVIM standards, although a great variability in measurements prevented the authors from recommending the NIBP device for therapeutic guidance [3]. In this study, the minimal number of eight animals for comparison with intra-arterial methods [11] was accomplished. The power analysis revealed that 20 animals were needed to detect a difference between two blood pressure measurement methods. This number was not reached for two reasons. First, the number of 16 horses was dictated by the surgical trial. Nevertheless, the present experimental setting provided the opportunity for additional CO measurements and pharmacological manipulation of blood pressure which is beyond the scope of studies in clinical patients. Secondly, five horses could not be fully instrumented. Therefore, the results of this study should be interpreted carefully. Moreover, it needs to be considered that consensus validation standards in veterinary medicine are lacking [26] and that ACVIM recommendations were developed for conscious dogs and cats.

One limitation of our study is the low number of measurements in the hypotensive and hypertensive blood pressurestates. Considering critical blood pressures, results should, therefore, be interpreted carefully. Large-scaled studies with heterogenous groups of horses and naturally occurring hyper- and hypotension might help to evaluate the source of the inaccuracy.

Regardless of the quality of oscillometric curves displayed by HDO, all data were included in the statistical analysis. Since artefacts are claimed to be filtered by HDO and should not influence measurements [4], it is unlikely that curve quality altered results. Moreover, it is important to be able to rely on data even if HDO software for curve analysis might be unavailable.

The blood pressure transducers were not calibrated against a mercury manometer, and standard electronic pressure transducers were preferred over microtransducer-tipped catheters to better reflect practical clinical standards, which can be regarded as a limitation of the study.

To minimise the influence of individual artery wall properties or blood flow characteristics, ideally, the same artery for IBP and NIBP measurement is used. However, repetitive occlusion of the MA artery by the HDO cuff was very likely to interfere with IBP measurements.

## 5. Conclusions

Agreement of the HDO monitor with the cuff placed over a metatarsal artery led to an acceptable agreement with IBP measurements for MAP in normotensive anaesthetised horses. However, during hypotension and hypertension, measurements were not valid, which is associated with the risk of clinically relevant hypotension going unnoticed.

Consequently, IBP remains preferable for clinical decision making in haemodynamically unstable patients. Overall, determination of MAP by HDO performed better than detection of DAP and SAP. Moreover, poor oscillometric signal quality may indicate the presence of hypotension and warrants further investigation. In this study, the HDO monitor failed to meet most of the American College of Veterinary Internal Medicine validation criteria.

## Figures and Tables

**Table 1:** Compliance with the American College of Veterinary Internal Medicine (ACVIM) consensus guidelines. Comparison of the invasively (IBP) and non-invasively (high-definition oscillometry (HDO)), measured systolic arterial pressure (SAP), diastolic arterial pressure (DAP), and mean arterial pressure (MAP) obtained at the metatarsal artery (MA) in 11 anaesthetised horses with validation criteria for blood pressure devices proposed by the ACVIM consensus statement [11].

Parameters	SAP	DAP	MAP	2007 ACVIM Consensus Statement
Mean difference of paired measurements (mmHg)	21.3	19.6	13.9	≤±10
Standard deviation (mmHg)	15.1	15.2	12.1 *	≤15
Correlation between paired measurements	0.8	0.73	0.81	≥0.9
Number measurements within 10 mmHg of reference method (%)	25	29.6	51.3 *	≥50
Number of measurements within 20 mmHg of reference method (%)	55.9	64.5	71.1	≥80
Minimal number of animals for comparison with intra-arterial method	11 *			8

* = measurements meeting criteria of ACVIM standards.

## Data Availability

The datasets analysed during the current study are available from the corresponding author on reasonable request.

## Supplementary Materials

The following are available online at https://www.mdpi.com/article/10.3390/ani12030363/s1, Table S1: Cardiovascular-derived variables for the calculation of cardiac index and systemic vascular resistance index.
