# Supplementary material for: Agreement of High-Definition Oscillometry (HDO) and Invasive Blood Pressure Measurements at a Metatarsal Artery in Isoflurane-Anaesthetised Horses
Source: Animals (Basel). 2022 Feb 2;12(3):363. doi: 10.3390/ani12030363 (PMC8833836; doi:10.3390/ani12030363)
Supplement: Supplementary file 1 [file animals-12-00363-s001.zip › animals-1573072-supplementary.pdf]

Table S1. Cardiovascular derived variables for the calculation of cardiac index and systemic vascular resistance index.

**Variable**	**Equation**	**Units**
Cardiac index (CI)	$CI = \frac{CO}{BW}$	mL/min/kg
Systemic vascular resistance index (SVRI)	$SVRI = \frac{MAP - CVP}{CI} x\ 80$	dynes sec^{-1} cm^{-5} kg^{-1}

BW, body weight; CO, cardiac output; CVP, central venous pressure; MAP, mean arterial pressure
